# Age-Dependent Skeletal Muscle Mitochondrial Response to Short-Term Increased Dietary Fructose

**DOI:** 10.3390/antiox12020299

**Published:** 2023-01-28

**Authors:** Cristina Gatto, Angela Di Porzio, Raffaella Crescenzo, Valentina Barrella, Susanna Iossa, Arianna Mazzoli

**Affiliations:** Department of Biology, University of Naples Federico II, Complesso Universitario Monte Sant’Angelo, Via Cinthia, 80126 Naples, Italy

**Keywords:** fructose, skeletal muscle, mitochondria, oxidative stress, proton leak, insulin resistance

## Abstract

The harmful effect of a long-term high-fructose diet is well established, but the age-dependent physiological responses that can be triggered by a short-term high-fructose diet in skeletal muscles have not been deeply explored. Therefore, the aim of this work was to compare the alterations in mitochondrial energetic and insulin responsiveness in the skeletal muscle induced by a short-term (2 weeks) fructose feeding in rats of different ages. For this purpose, fructose and uric acid levels, insulin sensitivity, mitochondrial bioenergetics and oxidative status were evaluated in the skeletal muscles from young (30 days old) and adult (90 days old) rats. We showed that, even in the short term, a high-fructose diet has a strong impact on skeletal muscle metabolism, with more marked effects in young rats than in adults ones. In fact, despite both groups showing a decrease in insulin sensitivity, the marked mitochondrial dysfunction was found only in the young rats, thus leading to an increase in the mitochondrial production of ROS, and therefore, in oxidative damage. These findings underscore the need to reduce fructose consumption, especially in young people, to preserve the maintenance of a metabolically healthy status.

## 1. Introduction

Fructose, present in small quantities in fruit and honey, has always been used in human diets, but between the past 10 and 20 years its utilization has increased enormously, and this event has been linked to a rise in obesity and metabolic disorders [1]. This represents a huge issue for public health, not only for the adult population, but also, and above all, for the young population, since children prefer sweeter food and beverages compared to adults [2]. The amount of fructose consumption is highest in adolescents aged 12–18 and it provides, on average, 12% of their energy, with 25% of adolescents acquiring more than over 15% of their energy from consuming fructose [3]. It has been shown that the excessive consumption of added sugars increases the probability for overweight/obesity among youths [4], and high fructose intake is also responsible for ectopic fat deposition and insulin resistance in metabolically relevant tissues, such as the skeletal muscle [5,6].

Mitochondrial function plays an important role in metabolic health, especially in the skeletal muscles [7]. In particular, a decrease in mitochondrial fatty acid utilization in the skeletal muscles [8] can contribute to lipid accumulation that could generate toxic metabolites, such as acyl-CoA, diacylglycerol and ceramides. In addition, since mitochondria are one of the major sources of reactive oxygen species (ROS) in the cells, an increase in oxidative stress linked to mitochondrial dysfunction has been proposed to contribute to insulin resistance in skeletal muscles [9,10]. In agreement with the above hypothesis, several studies showed a decrease in the number and function of mitochondria in the skeletal muscles of type 2 diabetic patients [9,11,12], although other studies have indicated that insulin resistance can be dissociated from mitochondrial impairment [13,14].

Based on the above findings regarding the highest rate of consumption of fructose by young populations and considering the largely different metabolic and physiological profiles between young people and adults, the investigation of the metabolic consequences induced by a fructose-rich diet in these two populations becomes relevant. We have already demonstrated that short-term fructose intake elicits no increase in the amount of body lipids in young rats, however, they developed hepatic insulin resistance and oxidative stress [15], as well as inflammation, oxidative stress and mitochondrial impairment in the hippocampus [16]. Therefore, the major aim of this study was to compare the alterations in mitochondrial energetic and insulin responsiveness in the skeletal muscles induced by a short-term (2 weeks) high-fructose feeding in rats of different ages: young (30 days old) and adult (90 days old) ones.

In detail, at the end of the dietary treatment, skeletal muscle insulin signaling, the degree of oxidative stress in both the skeletal muscle homogenates and mitochondria, as well as the mitochondrial function were evaluated after short-term (2 weeks) high-fructose feeding in rats of different ages.

## 2. Materials and Methods

### 2.1. Experimental Design

Male Sprague Dawley rats (Charles River, Italy) of 30 (young) or 90 (adult) days of age were caged in a temperature-controlled room (23 ± 1 °C) with a 12 h light/dark cycle (06.30–18.30). Both the young and adult rats were fed for 2 weeks a fructose-rich (young-F and adult-F) or control (young-C and adult-C) diet. The composition of the two diets is shown in Table 1. At the end of the experimental period, the rats were anesthetized with sodium pentothal (40 mg kg^−1^ i.p.) and euthanized by decapitation, and their hind leg skeletal muscles were collected. The experimental animal procedures were all approved by “Comitato Etico-Scientifico per la Sperimentazione Animale” of the University of Naples Federico II and authorized by Italian Health Minister (260/2015-PR). This work observes all of the animal ethics principles and regulations of the Italian Health Ministry. The authors assured that all of the steps were taken to minimize the pain and suffering of the animals. 

### 2.2. Glucose Tolerance Test

The glucose tolerance test was carried out a day before the euthanasia after a 6 h fasting period starting from 8 a.m. Our choice of the fasting duration was based on the concern expressed by several recent studies that overnight fasting in rodents is not ideal because as they are nocturnal animals, they consume most of their daily calories overnight. This, combined with their relatively high metabolic rate, means that an overnight fast is a relatively long time for the animals to be deprived of food, and it may induce a state that is more similar to starvation than to an overnight fast [17,18]. After 6 h fasting, blood samples were collected from the tail veins in tubes with EDTA for plasma separation, and subsequently, 2 g kg^−1^ of glucose was injected intraperitoneally, and blood samples were taken after 20, 40, 60, 90 and 120 min and centrifuged at 4 °C for 8 min at 1400× *g*. For a further determination of glucose and insulin, plasma aliquots were collected and stored at −20 °C. The plasma glucose concentration was determined using a colorimetric enzymatic method, while the plasma insulin concentration was evaluated using an ELISA kit in a single assay to avoid interassay variations. Skeletal muscle insulin sensitivity was determined according to Abdul-Ghani et al. [19]. 

### 2.3. Skeletal Muscle Composition

Skeletal muscle homogenates were obtained using a 50 mM phosphate buffer with a pH of 7.0 and used to evaluate skeletal muscle composition. 

Fructose, uric acid and triglycerides levels were assessed using colorimetric enzymatic. Ceramide content was measured by ELISA, as previously reported [15], while tissue glycogen was assessed by performing a direct enzymatic procedure [20].

### 2.4. Fatty Acid Synthase Activity

Fatty acid synthase (FAS) activity was evaluated in skeletal muscle homogenates in a buffer containing Tris 10 mM, KCl 175 mM, pH 7.5 (1:8 *w/v*), as described previously [21]. 

### 2.5. Preparation of Skeletal Muscle Isolated Mitochondria 

Mitochondria were isolated in the hind leg muscles, which after the removal of excess connective tissue, were minced finely and washed in a medium containing Tris 50 mM, KCl 100 mM, pH 7.5, MgCl_2_ 5 mM, EDTA 1 mM, EGTA 5 mM and 0.1 % (*w/v*) fatty-acid-free BSA and treated for 4 min with protease nagarse. Then, tissue fragments were repeatedly washed and homogenized 1:8, *w/v* with the above medium at 500 rpm (4 strokes/min). Homogenates were centrifuged at 3000× *g* for 10 min, and the pellet was resuspended and centrifuged at 500× *g* for 10 min. The supernatant was then centrifuged at 3000× *g* for 10 min, and the pellet was washed once and resuspended in a medium containing Tris 50 mM, sucrose 250 mM, pH 7.5 and 0.1% fatty-acid-free BSA. 

### 2.6. Oxidative Stress Parameters

Skeletal muscle homogenates were used to evaluate the oxidative stress markers.

Lipid peroxidation was measured both in the isolated mitochondria and in the skeletal muscle homogenates according to Fernandes et al. [22].

The superoxide dismutase (SOD) activity was evaluated both in the skeletal muscle homogenates and in the isolated mitochondria according to Flohè and Otting [23], while the catalase activity was determined only in the skeletal muscle homogenates according to Maehly and Chance [24]. 

The xanthine oxidase (XO) activity was evaluated according to Cos P. et al. [25], while the NADPH oxidase activity was measured using the method of Bettaieb et al., which we changed [26]. Briefly, 1:10 *w/v* tissues were homogenized in Krebs buffer (ice-cold) and centrifuged for 10 min at 800× *g* and 4 °C. The supernatant was then centrifuged for 2 h at 30,000× *g* and 4 °C. Then, the membrane fraction contained in the pellet was suspended in Krebs buffer, and the protein concentration was measured. Krebs buffer with 500 μM of NADPH was added to aliquots containing 100 μg of protein, and changes in absorbance at 340 nm were measured for 10 min at 30 s intervals. 

### 2.7. Mitochondrial Oxidative Capacities, Marker Enzymes and Proton Leak Kinetics in Skeletal Muscles

Mitochondrial oxidative capacities were assessed by polarographically measuring the oxygen consumption rate in a 3 mL glass cell at 30 °C using a Clark type electrode (Yellow Springs Instruments, Yellow Springs, OH, USA). Mitochondria isolated from skeletal muscle were incubated in a medium containing MgCl_2_ 6 mM, KCl 30 mM, sucrose 75 mM, KH_2_PO_4_ 20 mM, EDTA 1 mM and 0.1% (*w/v*) fatty-acid-free BSA at pH 7.0. The substrates used were glutamate 10 mM + malate 2.5 mM, succinate 10 mM + rotenone 3.8 mM and malate 2.5 mM + palmitoyl-carnitine 40 mM. The measurements were carried out in the presence of ADP 0.3 mM to evaluate respiratory capacity linked to ATP synthesis or after the addition of carbonyl cyanide 4-(trifluoromethoxy) phenylhydrazone (FCCP) 1 mM with succinate + rotenone as the substrate to evaluate maximal uncoupled respiratory capacity.

The Cytochrome Oxidase (COX) activity was measured in isolated mitochondria that were incubated for 30 min at 4 °C after the addition of 0.1 g/10 mL lubrol. The measurement was carried out polarographycally at 37 °C. 

Citrate Synthase (CS) activity was determined according to Srere [27] in the isolated mitochondria. 

To assess the kinetic response of the proton leak to changes in the membrane potential, we titrated the activity of the respiratory chain with inhibitors in the presence of oligomycin to prevent ATP synthesis and obtained the resulting titration curve of membrane potential against respiration rate. Mitochondrial oxygen consumption and mitochondrial membrane potential were measured as previously described [28]. 

### 2.8. Western Blotting of Skeletal Muscle Proteins 

Aliquots of both the homogenates and the isolated mitochondria from the skeletal muscles (20 μg of proteins) were denatured in Laemmli’s buffer and loaded on a 10% SDS–polyacrylamide gel. After the run, the gels were transferred to PVDF (polyvinylidene difluoride) membranes for 90 min at 0.8 mA/cm^2^. The membranes were preblocked in a blocking buffer (BB: PBS, bovine albumin serum 3%, Tween 20 0.3%) for 1 h and subsequently incubated overnight at 4 °C with antibodies against phospho-(Ser473)-Akt1 (p-Akt) (1:1000 in BB), phospho-(Ser9)-glycogen synthase kinase 3β (p-GSK) (1:1000 in BB), GLUT-4 (1:200 in BB), GLUT-5 (0.5 μg/mL in BB), UCP-3 (1:1000 in BB), Oxphos (1:250 in BB) and ANT (1:100 in BB). The membranes were washed and incubated for 1 h at RT with horseradish peroxidase-conjugated secondary antibodies (1:5000 in BB for p-Akt, p-GSK, GLUT-4, GLUT-5, UCP-3 and ANT or 1:100 000 for anti-Oxphos). The membranes were washed and incubated at RT with Immobilon chemiluminescent substrate, for p-Akt, p-GSK, GLUT-4 and GLUT-5, or with the Excellent Chemiluminescent detection Kit for Oxphos, UCP-3 and ANT. For the loading control, the membranes were stripped for 30 min at 50 °C and incubated overnight at 4 °C with Akt1 polyclonal antibody (1:1000 in BB) to normalize the p-Akt signal, GSK-3β monoclonal antibody (1:1000 in BB) to normalize the p-GSK signal, while the actin polyclonal antibody (1:1000 in BB) was used to normalize the Akt1, GSK-3b, GLUT-4 and GLUT-5 signal. Voltage-dependent anion selective channel proteins 1 (VADC1) and Ponceau red staining were used to normalize the UCP-3, ANT and Oxphos signals. Image Lab Software was used to densitometrically quantify the chemidoc images of the bands. 

### 2.9. Statistical Analysis

Data are reported as mean values ± SEM. Graph Pad Prism 9 (GraphPad Software, San Diego, CA, USA) was used to confirm that raw data have a normal distribution and to perform two-way ANOVA followed by the Tukey post hoc test. In all of the analyses, the probability of <5% (*p* < 0.05) was considered to be statistically significant. 

### 2.10. Materials

Salts, buffers and Bovine serum albumin fraction V (BSA) were purchased from Sigma-Aldrich (St. Louis, MO, USA). Colorimetric enzymatic kits were purchased from Sigma Aldrich (St. Louis, MO, USA) for fructose, GS Diagnostics SRL (Guidonia Montecelio, Rome, Italy) for glucose and uric acid and SGM Italia (Rome, Italy) for triglycerides. Elisa kit for insulin determination was purchased from Mercodia AB (Uppsala, Sweden). Chemiluminescent substrate, Immobilon (Millipore Corporation, Billerica, MA 01821, USA), Excellent Chemiluminescent detection Kit (ElabScience, Microtech, Naples, Italy), Polyvinylidene difluoride (PVDF) membrane (Millipore, Billerica, MA, USA) and dye reagent for protein titration (Bio-Rad, Hercules, CA, USA) were used for Western blotting. The antibodies used for Western blot analysis were purchased by Cell Signaling (Danvers, MA, USA) for p-Akt and Akt1, Santa Cruz Biotechnology (Santa Cruz, CA, USA) for p-GSK, GSK-3β, GLUT-4 and VDAC1, Invitrogen (Carlsbad, CA, USA) for GLUT-5, Calbiochem (San Diego, CA, USA) for UCP-3, Abcam, (Cambridge, UK) for Oxphos, Biogenesis Ltd. (England, UK) for ANT, Sigma-Aldrich (St Louis, MO, USA) for actin.

## 3. Results

### 3.1. Skeletal Muscle Insulin Signaling

**Figure 1:**
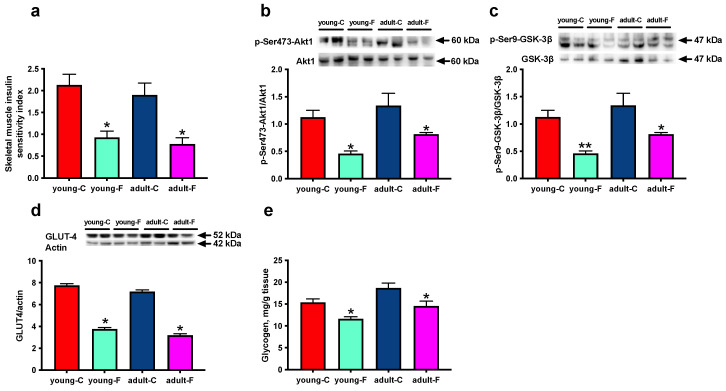
Skeletal muscle insulin signalling. (**a**) Skeletal muscle insulin sensitivity index, Western blot quantification (with representative Western blots) of (**b**) p-Akt, (**c**) p-GSK, (**d**) glucose transporter 4 (GLUT-4) and (**e**) content of glycogen in skeletal muscle in young and adult rats fed a control (young-C and adult-C) or fructose-rich diet (young-F and adult-F) for 2 weeks. Values are the means ± SEM of eight different rats. * *p* < 0.05, ** *p* < 0.01 compared to respective controls (two-way ANOVA followed by Tukey post-test).

As shown in Figure 1a, the skeletal muscle insulin sensitivity index was significantly lower in the fructose-fed rats compared to that of the respective controls, both in the young and adult animals. At the molecular level, we observed significant changes in the main downstream effectors of insulin signalling in the skeletal muscles, namely Akt1 (Figure 1b) and GSK-3β (Figure 1c). In detail, the lower abundance of the active, phosphorylated form of Akt is indicative of a downregulation of the Akt1-linked signal transduction in the fructose-fed rats compared to that of their respective controls, which is in line with the decrease in the level of insulin-sensitive GLUT-4 (Figure 1d). Similarly, the lower abundance of the inactive, phosphorylated form of GSK-3β implies an inhibition of the activity of glycogen synthesis, which is confirmed by the decrease in the glycogen content (Figure 1e) in the fructose-fed rats compared to that of the respective controls. The Akt1/actin and GSK3β/actin ratios have been evaluated as well, and no changes were found between the four experimental groups, thus confirming that the decreased phosphorylation of both of the proteins reflects a decreased activation (Figure S1a,b).

### 3.2. Skeletal Muscle Composition

**Figure 2:**
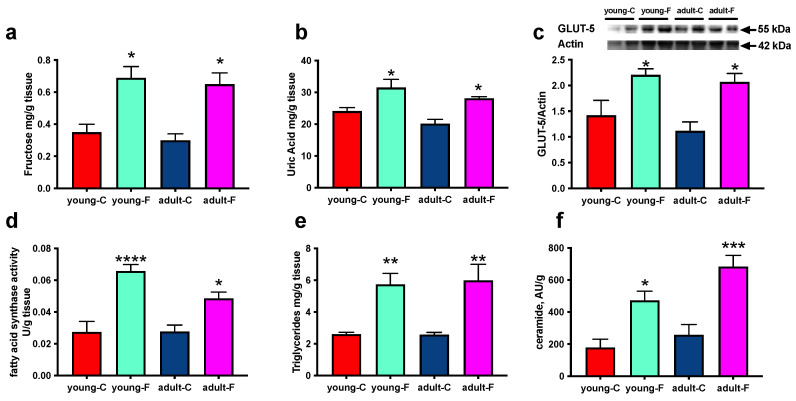
Composition of skeletal muscle (**a**) Content of fructose, (**b**) uric acid, (**c**) glucose transporter 5 (GLUT-5), (**d**) fatty acid synthase (FAS) activity, (**e**) triglycerides and (**f**) ceramide in skeletal muscles of young and adult rats fed a control (young-C and adult-C) or fructose-rich diet (young-F and adult-F) for 2 weeks. Values are the means ± SEM of eight different rats. * *p* < 0.05, ** *p* < 0.01, *** *p* < 0.001, **** *p* < 0.0001 compared to respective controls (two-way ANOVA followed by Tukey post-test).

The skeletal muscle content of fructose and uric acid, its main metabolites, were significantly higher both in the young-F rats and the adult-F rats, which is in agreement with the increase in the specific fructose transporter GLUT-5 (Figure 2a–c) in the same groups compared with that of the respective controls. FAS activity, as along with triglycerides and ceramide in the skeletal muscle, was found to be increased in both the young and adult rats fed a fructose-rich diet (Figure 2d–f). 

### 3.3. Skeletal Muscle Oxidative Status

**Figure 3:**
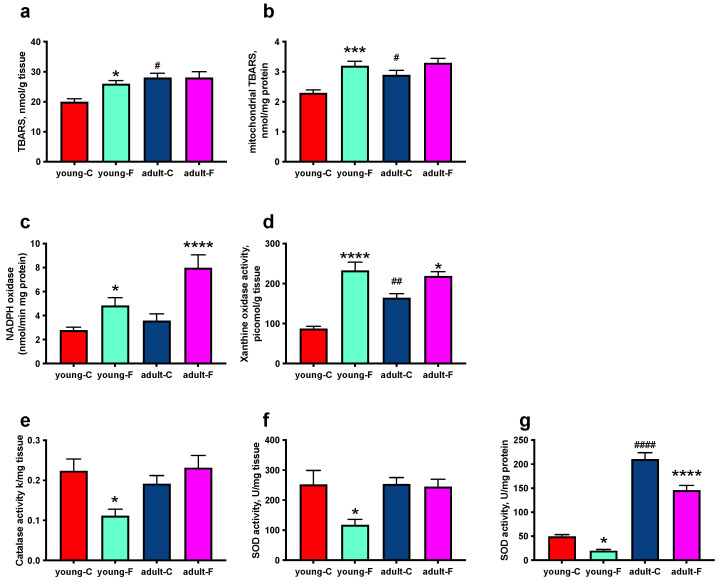
Markers of oxidative status. (**a**) Lipid peroxidation in skeletal muscle homogenates and (**b**) isolated mitochondria, (**c**) activities of NADPH oxidase, (**d**) xanthine oxidase, (**e**) antioxidant enzymes catalase, (**f**) cellular superoxide dismutase (SOD) and (**g**) mitochondrial SOD in young and adult rats fed a control (young-C and adult-C) or fructose-rich diet (young-F and adult-F) for 2 weeks. Values are the means ± SEM of eight different rats. * *p* < 0.05, *** *p* < 0.001, **** *p* < 0.0001 compared to respective controls; # *p* < 0.05, ## *p* < 0.01, #### *p* < 0.0001 compared to young-C rats (two-way ANOVA followed by Tukey post-test). TBARS = thiobarbituric acid related substances.

As a marker of oxidative damage to the lipids, we evaluated the lipid peroxidation, and the obtained results indicated an increase in the level of dietary fructose only in young-F rats both in the skeletal muscle homogenates and in the isolated mitochondria, as well as more oxidative damage in the adult rats compared to that in the young ones (Figure 3a,b). The activities of two oxidant-producing enzymes, namely NADPH oxidase and xanthine oxidase, were upregulated in the young and adult rats fed a fructose-rich diet, with an age-associated increase only in xanthine oxidase activity (Figure 3c,d). As for the antioxidant system capacity, the catalase activity decreased after 2 weeks of fructose feeding only in the young rats, while no difference was found in the adults (Figure 3e). In addition, the SOD activity, which was evaluated in skeletal muscle homogenates, exhibited a diet-induced decrease only in the young rats (Figure 3f), while the mitochondrial SOD activity was lower both in the young and adult rats after being fed the fructose diet, and interestingly, it was about four-fold higher in the adult rats compared to that in the young ones (Figure 3g).

### 3.4. Mitochondrial Enzymes and Oxygen Consumption

**Figure 4:**
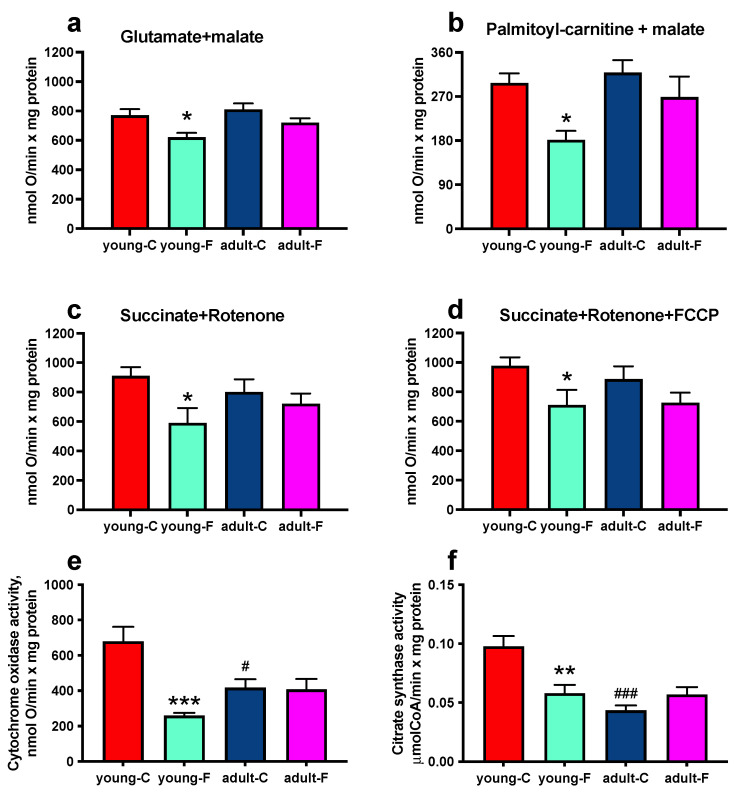
Mitochondrial markers. (**a**–**d**) Mitochondrial oxygen consumption and activities of (**e**) cytochrome oxidase and (**f**) citrate synthase measured in skeletal muscle isolated mitochondria of young and adult rats fed a control (young-C and adult-C) or fructose-rich diet (young-F and adult-F) for 2 weeks. Values are the means ± SEM of eight different rats. * *p* < 0.05, ** *p* < 0.01, *** *p* < 0.001 compared to respective controls; # *p* < 0.05, ### *p* < 0.001 compared to young-C rats (two-way ANOVA followed by Tukey post-test).

The mitochondrial oxidative capacities in phosphorylating conditions were evaluated in mitochondria isolated from skeletal muscles using NAD, FAD and lipid substrates, and the results indicated a more significant decrease in the young-F rats compared to that in the young-C rats in the presence of glutamate, palmitoyl-carnitine or succinate (Figure 4a–c), while no significant variation was found in the adult rats. A similar decrease was also found in the young-F rats when succinate-supported respiration was measured in the uncoupled conditions (Figure 4d). 

**Figure 5:**
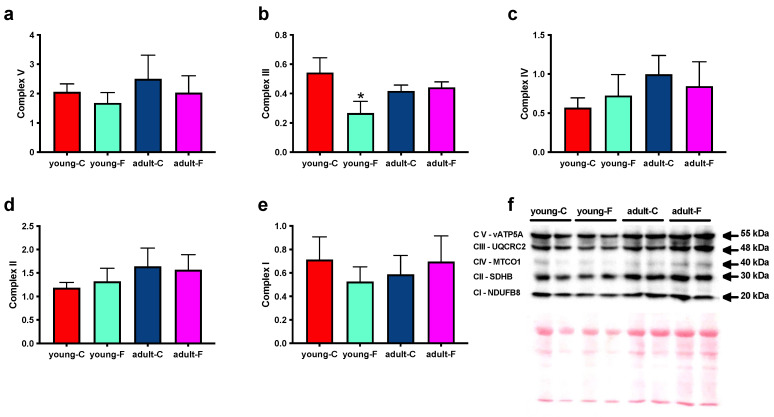
Mitochondrial complexes. (**e**) Complex I, (**d**) complex II, (**b**) complex III, (**c**) complex IV, and (**a**) complex V, together with representative Western blots, which were normalized via Ponceau staining (**f**), assessed in isolated mitochondria from young and adult rats fed a control (young-C and adult-C) or fructose-rich diet (young-F and adult-F) for 2 weeks. Values are the means ± SEM of eight different rats. * *p* < 0.05 compared to respective controls (two-way ANOVA followed by Tukey post-test).

We also assessed the activity of two mitochondrial marker enzymes, CS, which is located in the mitochondrial matrix, and COX, which is located in the inner mitochondrial membrane. The COX and CS activity levels (Figure 4e,f) are decreased in the isolated mitochondria from the young-F rats compared to that in their respective controls, while the fructose-rich diet had no effect on the activity of the two enzymes in adult rats, which exhibited an age-related lower CS activity level (Figure 4f). Moreover, we analyzed the abundance of respiratory complexes, since the modification of mitochondrial function could be a consequence of an altered number of proteins constituting the respiratory complexes. The analysis of the various components of the mitochondrial respiratory chain in the skeletal muscle (Figure 5 and Figure S2) showed no significant variation in complexes I, II, IV, and V in the fructose-fed rats compared to those in the controls (Figure 5a,c–e and Figure S2a,c–e) and a reduction in complex III in the young-F rats compared with that in the young-C rats (Figure 5b and Figure S2b).

### 3.5. Mitochondrial Proton Leak 

**Figure 6:**
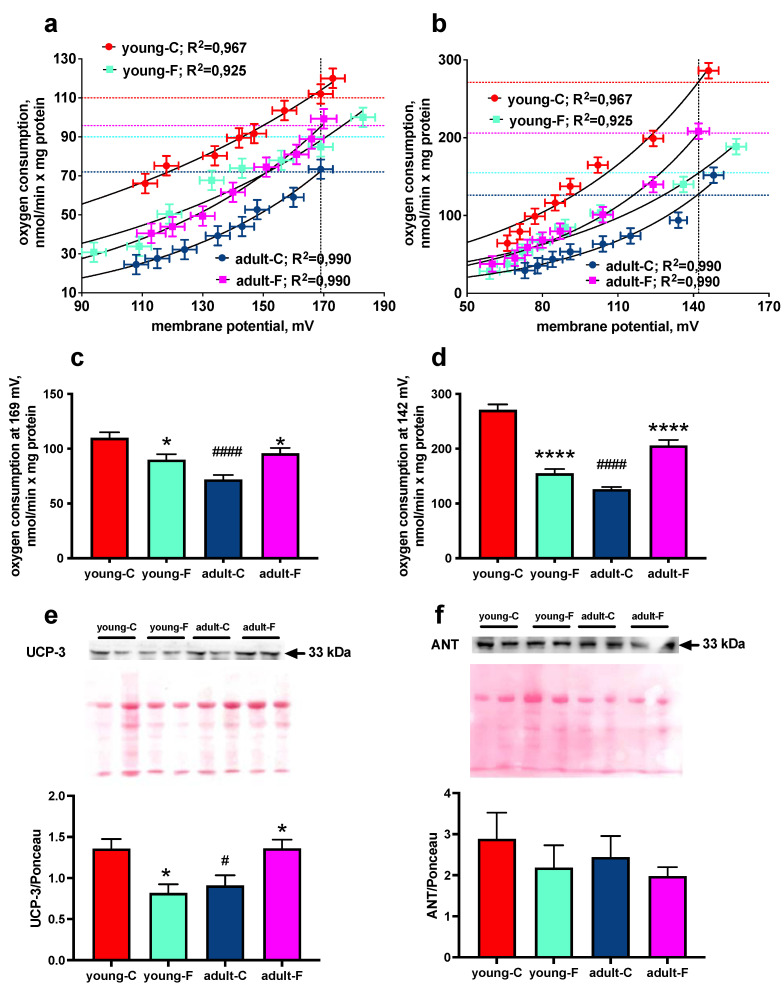
Mitochondrial proton leak. (**a**) Basal and (**b**) palmitate-induced proton leak kinetics, (**c**,**d**) oxygen consumption at the highest common membrane potential, (**e**) Western blot quantification via Ponceau staining (with representative Western blots) of UCP-3 and (**f**) ANT in isolated mitochondria from skeletal muscles of young and adult rats fed a control (young-C and adult-C) or fructose-rich diet (young-F and adult-F) for 2 weeks. Values are the means ± SEM of eight different rats. * *p* < 0.05, **** *p* < 0.0001, compared to respective controls; # *p* < 0.05, #### *p* < 0.0001 compared to young-C rats (two-way ANOVA followed by Tukey post-test).

The titration of the mitochondrial electron transport chain by malonate coupled with the simultaneous measurements of oxygen consumption and the membrane potential gives an estimate of the mitochondrial proton leak kinetics, since the steady-state oxygen consumption rate (i.e., proton efflux rate) in non-phosphorylating mitochondria is equivalent to the proton influx rate due to proton leaking both in the absence (basal proton leak, Figure 6a) and in the presence (palmitate-induced proton leak, Figure 6b) of the uncoupling effect of fatty acids. Significantly lower values of H^+^ permeability, which were quantified by determining the oxygen consumption at the highest common membrane potential with and without palmitate, were found in the fructose-fed young rats, while an opposite result was found in the fructose-fed adult rats (Figure 6c,d). In fact, the adult-F rats showed higher values of proton leakage with and without palmitate compared to the those of the adult-C rats (Figure 6c,d). In addition, a marked age-dependent effect was evident in the adult-C rats that exhibited a significantly lower proton leakage, both basal and palmitate-induced, compared to that of the young rats (Figure 6c,d).

The molecular determinants of mitochondrial proton leakage have been suggested to be UCP-3 and ANT. In agreement with the results regarding proton leakage, there were lower and higher amounts of UCP-3 content in the skeletal muscles of the young-F and adult-F rats, respectively, compared with those of their controls, as well as significantly higher levels of UCP-3 in the young compared to those in the adult rats (Figure 6e and Figure S3a). No diet- or age-related differences were found in the ANT content (Figure 6f and Figure S3b). 

## 4. Discussion

The central result of the present study is that even if it is limited to short periods, high fructose intake can induce age-dependent alterations in mitochondrial energetics, which is in parallel with the development of oxidative stress and insulin resistance.

At the whole-body level, adult and young rats fed the fructose-rich diet displayed reduced skeletal muscle insulin sensitivity (Figure 1). Since the glucose buffering capacity after glucose loading is primarily determined by the skeletal muscle metabolic response to insulin [29], downstream targets of the insulin signaling pathway, named *p*-Akt, p-GSK and GLUT-4, were evaluated in the skeletal muscle of fructose-fed rats, and significantly lower values were found in both in the young and adult rats compared with those of the respective controls. This result well correlates with the decrease in glycogen deposition found in this tissue, since the non-phosphorylated form of GSK3β is active, and this in turn causes phosphorylation, and hence, the inhibition of glycogen synthase, the rate limiting step in the process of glycogen synthesis [30]. 

Our present results also point to ceramide and triglycerides as major players in the blunted action of insulin in the skeletal muscles of fructose-fed rats; both of them were found to have significantly increased in the adult and young rats (Figure 2). In fact, it is well known that ceramide and diacylglycerol (DAG) act as mediators of insulin resistance via the inhibition of Akt1 phosphorylation [31,32]. From our present results it appears that fructose, which is uptaken by the specific transporter GLUT-5, whose levels are increased, can be converted into fatty acids due to the activation of the metabolic pathway of de novo lipogenesis [33], as indicated by the increased activity of the limiting enzyme FAS that we found in this study [34]. The neosynthesized fatty acids are then transformed in DAG and triglycerides and furnish the substrate for the synthesis of ceramide [35]. 

Another dangerous consequence of fructose accumulation in the skeletal muscles is its phosphorylation, and hence, the depletion of ATP, with higher levels of intracellular uric acid, the main metabolic byproduct of fructose that is found in both adult and young rats. Uric acid production is associated with oxidative stress [36], and indeed, we found an increase in the level of activity of xanthine oxidase in the fructose-fed rats, which we identified as a source of ROS in the skeletal muscles [37], as well as in the level of activity of another main cellular source of free radicals, namely NADPH oxidase (Figure 3). Quite unexpectedly, we evidenced increased lipid peroxidation in young fructose-fed rats, both in the isolated mitochondria and in the tissue homogenates, while no sign of oxidative stress was evident in the adult rats. In addition, an age-related increase in the rate of oxidative damage was found in the adult control rats, which is in line with the further age-dependent increase in xanthine oxidase activity. Since the oxidative damage is due not only to the increased production of ROS, but also to a decrease in the antioxidant defenses, we evaluated the activity of two enzymes of the antioxidant defense system, namely catalase and SOD, which are both located in the cytoplasm and in the mitochondria. Interestingly, we found a decreased level of activity of antioxidant enzymes in the young fructose-fed rats, while in the adult fructose-fed rats, only the mitochondrial SOD was decreased. The sugar-induced decrease in the antioxidant enzyme activity level has already been reported in the hearts of young (3 weeks old) rats after 2 weeks of sucrose intake [38]. We can speculate that in young rats, as also suggested by Girard A. et al. [39], the antioxidant enzymes are damaged by an increase in ROS production, which indeed emerges from the analysis of the mitochondrial function that is described in detail below. Another intriguing result was found for the control rats, which showed a marked increase in the amount of mitochondrial SOD due to increasing age. This result agrees with previous studies, which showed an increase in SOD activity in the livers of human subjects during aging [40] and that SOD2 protein expression is constantly and significantly increased in the rat’s liver during the aging process [41]. 

A central player in the generation of ROS, and hence, in the induction of cellular oxidative stress, is represented by the mitochondria, and skeletal muscle mitochondrial function has been linked to the onset of insulin resistance [42,43]. We therefore considered it to be of interest to evaluate the putative changes in mitochondrial function elicited by high fructose intake (Figure 4). The mitochondrial maximal oxidative capacity with FAD-, NAD- and lipid-linked substrates was found to be lower only in the young fructose-fed rats, thus suggesting an impairment of the rate-limiting steps along the mitochondrial respiratory chain that is common to both complex I- and II-linked oxidation. Lower respiratory rates were shown also in the presence of FCCP, an artificial uncoupler that dissociates the oxidation of substrates from the synthesis of ATP, thus allowing us to exclude an impairment of the ATP synthesis machinery. Since changes in the mitochondrial function could arise from altered activity levels and/or the amount of respiratory complexes, we analyzed the abundance of the respiratory complexes I–V (Figure 5), and we found a decrease in the protein amount in complex III, which is located at the crossroads of the electron transport chain, which receives electrons independently from complexes I and II and transfers them to cytochrome c via the Q cycle. In addition, although the amount of complex IV was found to be unaltered, the measured activity of COX was lower in the young fructose-fed rats, thus suggesting that also this step in the respiratory chain was impaired probably at the level of enzymatic activity, which could have been damaged by increased ROS production. The lowered complex III protein content might be an important and rate-determining step that is responsible for the alterations of the mitochondrial oxidative metabolism in the young fructose-fed rats. Moreover, the reduced functionality of complex III can contribute to the enhanced oxidative damage found in these rats. In fact, it is well known that this respiratory complex contributes to ROS production in the mitochondria into either the matrix or the intermembrane space [44,45], and when the electron flux through this complex is lowered, it remains in a more reduced state, thus increasing the potential escape of electrons out of the respiratory chain to directly interact with oxygen. Similar results have been obtained in failing hearts 2 weeks after a myocardial infarction, where mitochondrial respiration was impaired and complex III was the primary site of metabolic dysfunction in mitochondria, and it was associated with increased ROS generation [46].

ROS production is also regulated by the degree of coupling between oxygen consumption and ATP synthesis. As a matter of fact, mitochondrial proton leakage is considered to be an important factor in the efficiency of energy conversion in several tissues (including the skeletal muscle), and it is well known that fatty acids can act as natural uncouplers of oxidative phosphorylation by generating a fatty acid-dependent proton leak pathway, which is a function of the amount of unbound fatty acids in the cell [47,48]. We therefore assessed whether the metabolic alterations that we found in this study induced by the fructose diet could be linked to altered skeletal muscle mitochondrial proton leakage (Figure 6). It is extremely interesting to notice that both the rates of basal and palmitate-induced proton leakage in the skeletal muscles were significantly increased due to fructose diet in the adult rats, while they were significantly decreased in the young fructose fed-rats. The above results are really intriguing since the degree of proton leakage deeply influences the ROS production. In fact, in the young fructose-fed rats, the decreased mitochondrial proton leakage rate, together with the reduction of mitochondrial SOD activity, determines an increase in ROS production, thus explaining the increased lipid peroxidation found in these rats. On the other hand, in adult fructose-fed rats, the increased rate of proton leakage gives rise to decreased ROS production that balances the decreased mitochondrial SOD activity, thus substantiating the absence of a marker of oxidative stress. The opposite variation in mitochondrial proton leakage found in the young and adult rats well correlates with the content of UCP-3, which were found lower and higher, respectively in the young and adult rats. In fact, very recently it has been demonstrated that the overexpression of UCP-3 in the skeletal muscles is accompanied by increased muscle mitochondrial inefficiency in vivo [49]. In addition, it is well known that UCP-3 expression stimulation is a counterregulatory response that is elicited to avoid oxidative stress [50,51,52]. An interesting observation that can be drawn from the analysis of the data related to proton leakage is that this mitochondrial characteristic is a highly age-dependent one. In fact, in the young rats, the rate of proton leakage is higher compared to that in the adult rats, and its downregulation by fructose diet promotes ROS production. In the adult rats, the rate of proton leakage is lower, and the fructose-induced upregulation counteracts ROS production. Thus, by looking at the age effect, the skeletal muscle mitochondria from the young rats seem to be more protected by ROS production than those from the adult rats do, as also suggested by the marked lower mitochondrial SOD activity, UCP-3 content and degree of lipid peroxidation found in these rats. On the other hand, by looking at the fructose effect, we can speculate that adult skeletal muscle mitochondria better adapt to nutritional insult compared to the young skeletal muscle mitochondria. Therefore, the young rats seem to be more vulnerable to nutritional insults, probably because they have not yet reached a steady state typical of adulthood, while the adult rats are more capable of dealing with nutritional stimuli, at least on a short-term basis. In fact, we have previously administered a fructose-rich diet to adult rats for a longer (8 weeks) period, and differently from the present results, we have evidenced an increase in the mitochondrial coupling and oxidative stress concomitant with a decreased cellular SOD activity level [53]. Therefore, by comparing the present and previous results of the adult rats, an increased fructose intake elicits a compensatory response in the skeletal muscle mitochondria to avoid oxidative stress, which, however, is not maintained on a long-term basis, thus driving increased oxidative damage. Although no details of the long-term changes are available, it can be speculated that in the young rats, the deleterious effects seen in the adult rats after the long-term treatment appear much rapidly and are, therefore, detectable also after short-term treatment. Future studies will further investigate this issue.

It is of interest also to underline that the present results point to a strong link between the changes in mitochondrial efficiency and increased oxidative stress, while they do not support the general idea that mitochondrial dysfunction is involved in the onset of insulin resistance in the skeletal muscles. This probably arises from the fact that here we have studied the metabolic effects of fructose, which itself is able to stimulate de novo lipogenesis, therefore causing an increase in the cellular deposition of lipid metabolites both in young and adult rats independently from the changes in mitochondrial oxidation. Therefore, different nutritional insults can lead to similar metabolic imbalance, which, however, could arise from different molecular mechanisms.

## 5. Conclusions

In conclusion, the present results (summarized in Table 2) show that a short-term fructose-rich diet has a profound impact not only on the liver tissue, the main site of fructose metabolism, but also on another metabolically relevant tissue, the skeletal muscles. In addition, we were able to evidence that the side effects of increased fructose intake are more marked in young rats than they are in adult rats. In young rats, in fact, the unwanted consequence of the alteration in mitochondrial efficiency is the higher rate of mitochondrial ROS production, which could in turn exacerbate the direct effect of increased fructose delivery to the skeletal muscles. It is worth mentioning that this higher sensibility of young organisms to the damaging effect of fructose compared to those of the adult ones evidences that a strong effort should be devoted at reducing fructose intake, especially in adolescents, to promote a healthy adulthood. 

## Figures and Tables

**Table 1:** Experimental diets composition.

Component, g/100 g	Control Diet	Fructose Diet
Standard Chow ^a^	50.5	50.5
Sunflower Oil	1.5	1.5
Casein	9.2	9.2
Alphacel	9.8	9.8
Cornstarch	20.4	---
Fructose	---	20.4
Water	6.4	6.4
AIN-76 mineral mix	1.6	1.6
AIN-76 vitamin mix	0.4	0.4
Choline	0.1	0.1
Methionine	0.1	0.1
Energy content and composition		
Gross Energy Density (kJ/g)	17.2	17.2
ME content (kJ/g) ^b^	11.1	11.1
Proteins (% ME)	29.0	29.0
Lipids (% ME)	10.6	10.6
Carbohydrates (% ME)	60.4	60.4
Of which:		
Fructose	---	30.0
Starch	52.8	22.8
Sugars	7.6	7.6

^a^ 4RF21, Mucedola, Italy; ^b^ Estimated by computation using values (kJ/g) for energy content as follows: proteins, 16.736; lipids, 37.656; carbohydrates, 16.736. ME = metabolizable energy.

**Table 2:** Summary of results.

Parameter	Diet Effect		Age Effect
	Young	Adult	
**Insulin signaling**			
Skeletal muscle insulin sensitivity index	↓	↓	**ns**
Insulin signalling (pAkt, p-GSK, GLUT-4)	↓	↓	**ns**
Glycogen	↓	↓	↑
**Composition**			
Fructose, Uric Acid, GLUT-5	↑	↑	**ns**
Triglycerides and ceramide	↑	↑	**ns**
Fatty Acid Synthase	↑	↑	**ns**
**Redox homeostasis**			
TBARS	↑	**ns**	↑
Mitochondrial TBARS	↑	**ns**	↑
NADPH oxidase	↑	↑	**ns**
xanthine oxidase	↑	↑	↑
Catalase	↓	**ns**	**ns**
Superoxide dismutase	↓	**ns**	**ns**
Mitochondrial superoxide dismutase	↓	↓	↑
**Mitochondrial function**			
Activity of COX and CS	↓	**ns**	↓
State 3 respiration	↓	**ns**	**ns**
Complex III amount	↓	**ns**	**ns**
Basal proton leak	↓	↑	↓
Palmitate-induced proton leak	↓	↑	↓
UCP-3	↓	↑	↓
ANT	**ns**	**ns**	**ns**

## Data Availability

All data supporting the findings of this study are available within the article and its supplementary information files.

## Supplementary Materials

The following supporting information can be downloaded at: https://www.mdpi.com/article/10.3390/antiox12020299/s1, Figure S1: Western blot quantification (with representative Western blots) of (a) Akt1/actin ratio, (b) GSK-3β/actin ratio in skeletal muscles in young and adult rats fed a control (young-C and adult-C) or fructose-rich diet (young-F and adult-F) for 2 weeks. Values are the means ± SEM of eight different rats. Two-way ANOVA followed by Tukey post-test; Figure S2: (**e**) Complex I, (**d**) complex II, (**b**) complex III, (**c**) complex IV, and (**a**) complex V together with representative Western blot (f), normalized via VDAC1, assessed in isolated mitochondria from young and adult rats fed a control (young-C and adult-C) or fructose-rich diet (young-F and adult-F) for 2 weeks. Values are the means ± SEM of eight different rats. * *p* < 0.05 compared to respective controls (two-way ANOVA followed by Tukey post-test). Figure S3: Western blot quantification on VDAC1 (with representative Western blot) of UCP-3 (a) and ANT (b) in isolated mitochondria from skeletal muscles of young and adult rats fed a control (young-C and adult-C) or fructose-rich diet (young-F and adult-F) for 2 weeks. Values are the means ± SEM of eight different rats. * *p* < 0.05, compared to respective controls; # *p* < 0.05, compared to young-C rats (two-way ANOVA followed by Tukey post-test).
