# Supplementary material for: Age-Dependent Skeletal Muscle Mitochondrial Response to Short-Term Increased Dietary Fructose
Source: Antioxidants (Basel). 2023 Jan 28;12(2):299. doi: 10.3390/antiox12020299 (PMC9951991; doi:10.3390/antiox12020299)
Supplement: Supplementary file 1 [file antioxidants-12-00299-s001.zip › antioxidants-2141621-supplementary.pdf]

Supplementary Materials:

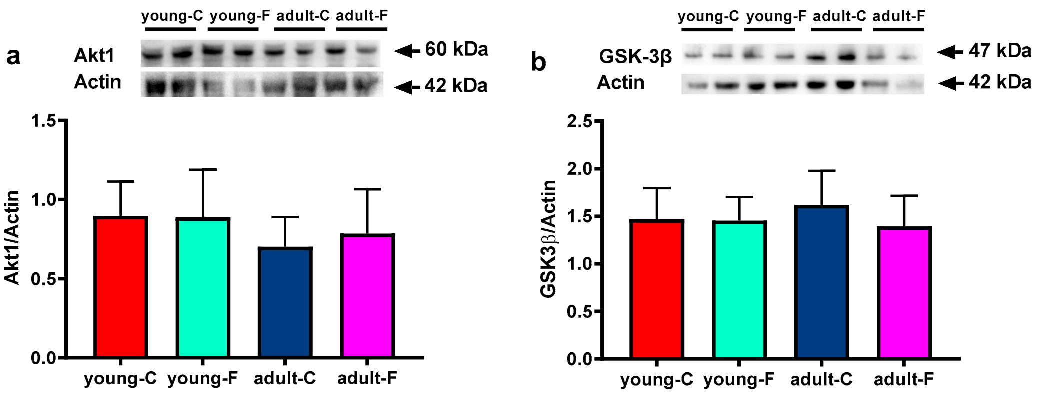

Figure S1. Western blot quantification (with representative Western blots) of (**a**) Akt1/actin ratio, (**b**) GSK-3β/actin ratio in skeletal muscles in young and adult rats fed a control (young-C and adult-C) or fructose-rich diet (young-F and adult-F) for 2 weeks. Values are the means ± SEM of eight different rats. Two-way ANOVA followed by Tukey post-test.

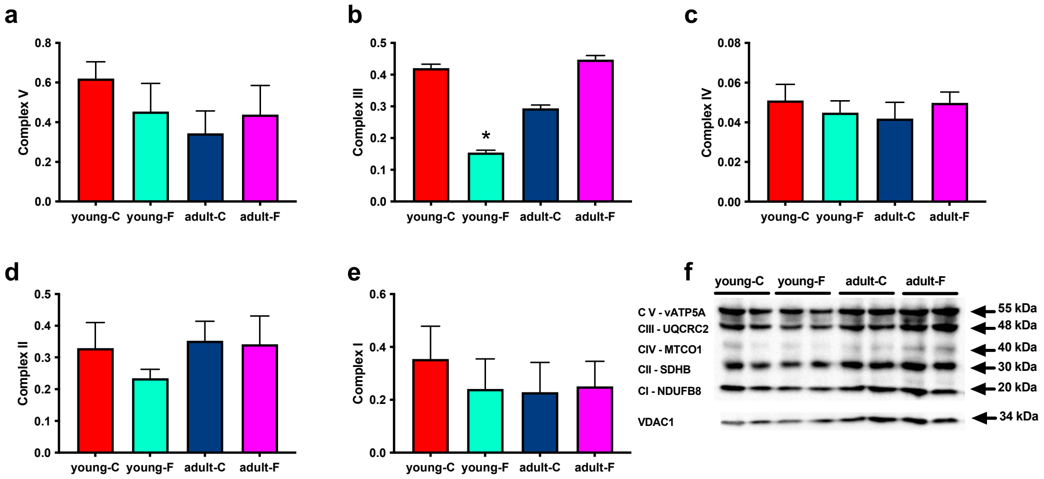

Figure S2. (**e**) Complex I, (**d**) complex II, (**b**) complex III, (**c**) complex IV, and (**a**) complex V together with representative Western blot (f), normalized via VDAC1, assessed in isolated mitochondria from young and adult rats fed a control (young-C and adult-C) or fructose-rich diet (young-F and adult-F) for 2 weeks. Values are the means ± SEM of eight different rats. * $p < 0.05$ compared to respective controls (two-way ANOVA followed by Tukey post-test).

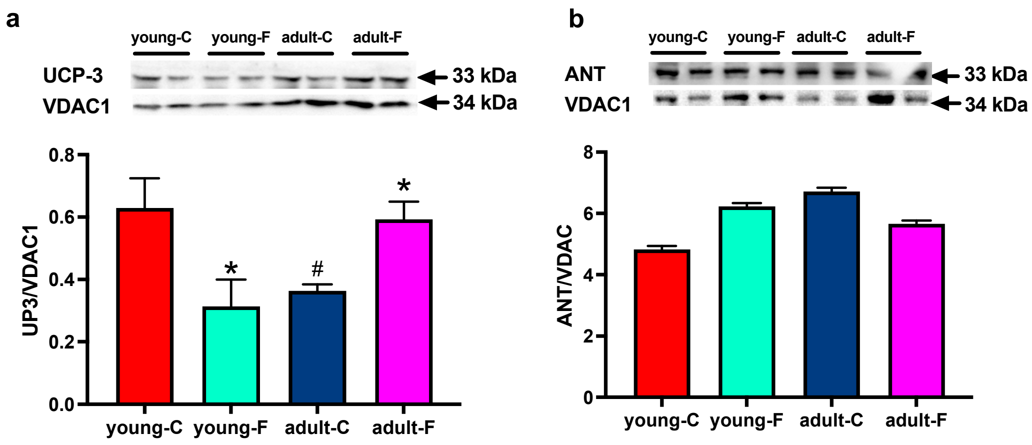

Figure S3. Western blot quantification on VDAC1 (with representative Western blot) of UCP-3 (a) and ANT (b) in isolated mitochondria from skeletal muscles of young and adult rats fed a control (young-C and adult-C) or fructose-rich diet (young-F and adult-F) for 2 weeks. Values are the means ± SEM of eight different rats. * $p < 0.05$, compared to respective controls; # $p < 0.05$, compared to young-C rats (two-way ANOVA followed by Tukey post-test).
